# 3D printing for heart valve disease: a systematic review

**DOI:** 10.1186/s41747-018-0083-0

**Published:** 2019-02-15

**Authors:** Volkan Tuncay, Peter M. A. van Ooijen

**Affiliations:** University of Groningen, University Medical Center Groningen, Hanzeplein 1, 9713GZ Groningen, The Netherlands

**Keywords:** Heart valves, Printing (three-dimensional), Stereolithography, Tomography (x-ray computed), Ultrasonography

## Abstract

**Background:**

Current developments showed a fast-increasing implementation and use of three-dimensional (3D) printing in medical applications. Our aim was to review the literature regarding the application of 3D printing to cardiac valve disease.

**Methods:**

A PubMed search for publications in English with the terms “3D printing” AND “cardiac valve”, performed in January 2018, resulted in 64 items. After the analysis of the abstract and text, 27 remained related to the topic. From the references of these 27 papers, 7 papers were added resulting in a total of 34 papers. Of these, 5 were review papers, thus reducing the papers taken into consideration to 29.

**Results:**

The 29 papers showed that about a decade ago, the interest in 3D printing for this application area was emerging, but only in the past 2 to 3 years it really gained interest. Computed tomography is the most common imaging modality taken into consideration (62%), followed by ultrasound (28%), computer-generated models (computer-aided design) (7%), and magnetic resonance imaging (3%). Acrylonitrile butadiene styrene (4/14, 29%) and TangoPlus FullCure 930 (5/14, 36%) are the most used printing materials. Stereolithography (40%) and fused deposition modeling (30%) are the preferred printing techniques, while PolyJet (25%) and laser sintering (4%) are used in a minority of cases. The reported time ranges from 30 min to 3 days. The most reported application area is preoperative planning (63%), followed by training (19%), device testing (11%), and retrospective procedure evaluation (7%).

**Conclusions:**

In most cases, CT datasets are used and models are printed for preoperative planning.

## Key points

- Computed tomography is the standard imaging modality for cardiac valve printing, followed by ultrasound, computer-aided design, and magnetic resonance imaging.
- Stereolithography and fused deposition modeling are the preferred methods for cardiac valve printing.
- Acrylonitrile butadiene styrene and TangoPlus FullCure 930 are the most used printing materials.
- The most reported application area is preoperative planning, followed by training, device testing, and retrospective procedure evaluation.

## Background

Although patient-specific three-dimensional (3D) visualisation already provides good insight into the complex anatomy of a patient, in some cases, this is not sufficient, and more advanced techniques are required, such as the use of virtual and augmented reality but also 3D printing [1, 2]. On the one side, 3D printing allows the surgeon to hold and examine the structures printed in a tactile way, sometimes providing a better insight into the 3D anatomy. On the other side, a real-life-size 3D printed anatomy allows to test procedures by introducing the actual implants, wires, and instruments into the printed anatomy.

The basis for 3D printing was laid in the 1980s. Medical applications arose from this new technology early on in the development, mainly in maxillofacial surgery. Although one paper already reported on the use of stereolithography (STL) printing of mitral valves based on ultrasound (US) imaging in patients as early as 2000 [3], the real interest for 3D printing in cardiovascular applications started some years later.

Building on the experience of the early adopters, the use of 3D printing recently has enormously increased in a wide variety of medical applications. The field has demonstrated itself as an example of multidisciplinary cooperation where radiologists, surgeons, and mechanical/biomedical engineers all provide their specific expertise in the different application areas [4]. These application areas vary from the printing of anatomical models for teaching and training [5] to models to inform the patient about treatment and from the preoperative evaluation of devices to the printing of guides and implants used during surgery. In recent years, cardiac anatomy and especially congenital heart disease have become one of the focus areas of 3D printing to easily visualise and explore complex cardiovascular anatomy. However, other applications that could have a major impact on the field of cardiothoracic surgery, such as planning of transcatheter aortic valve replacement (TAVR) and transcatheter mitral valve replacement (TMVR), are also arising. 3D printing can be used to tackle some of the challenges in these interventions such as patient selection, prosthesis choice and sizing, and innovation in valve design. In this narrative review, we discuss the current state of the art in this area from a technical point of view by considering the constraints and possibilities of the 3D printing technique based on published work that specifically focuses on 3D printing in cardiac valve disease treatment. We will look at general topics such as data preparation, time requirements, printer possibilities, and material properties relating to this specific application area. Possible clinical applications from the literature will also be introduced.

## Literature search

**Fig. 1:**
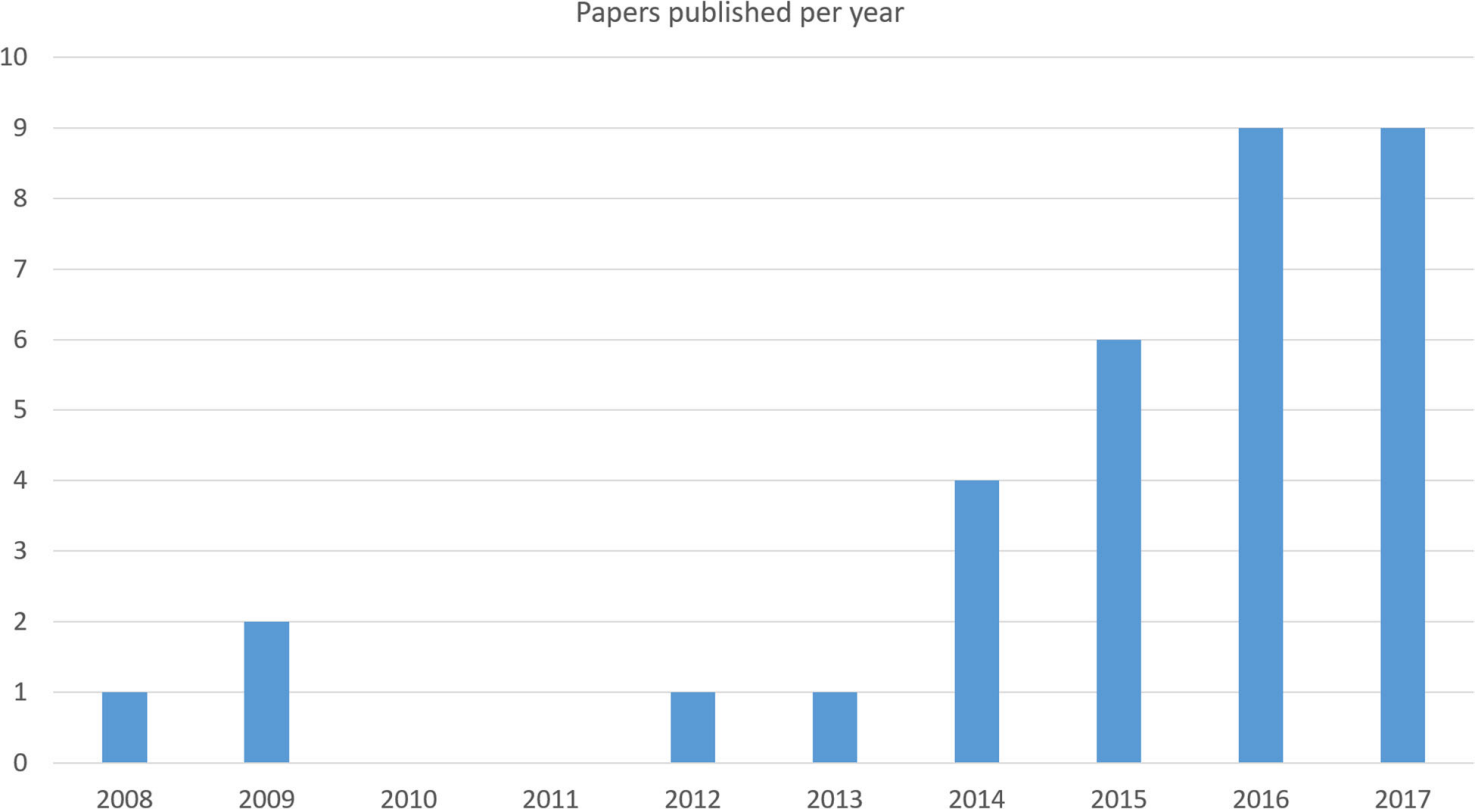
Number of publications on 3D printing in the application of cardiac valve assessment or replacement

A PubMed search for publications in English with the terms “3D printing” AND “cardiac valve” showed that interest in this topic is certainly gaining. It was performed in January 2018. Although our initial search resulted in 64 items, after the analysis of the abstracts and text, 27 remained valid and related to the review topic. From the references of these 27 papers, another 7 papers were added resulting in a total of 34 papers. Of these, five were earlier review papers, of which most only mentioned the specific case of 3D printing in cardiac valve diseases as a small subsection of their review, thus reducing the papers taken into consideration to 29. The 29 papers clearly showed that about a decade ago, the interest in 3D printing for this application area was emerging, but only in the past 2 to 3 years it really gained interest resulting in a steep increase in the number of publications (Fig. 1).

## Source data and pre-processing

**Fig. 2:**
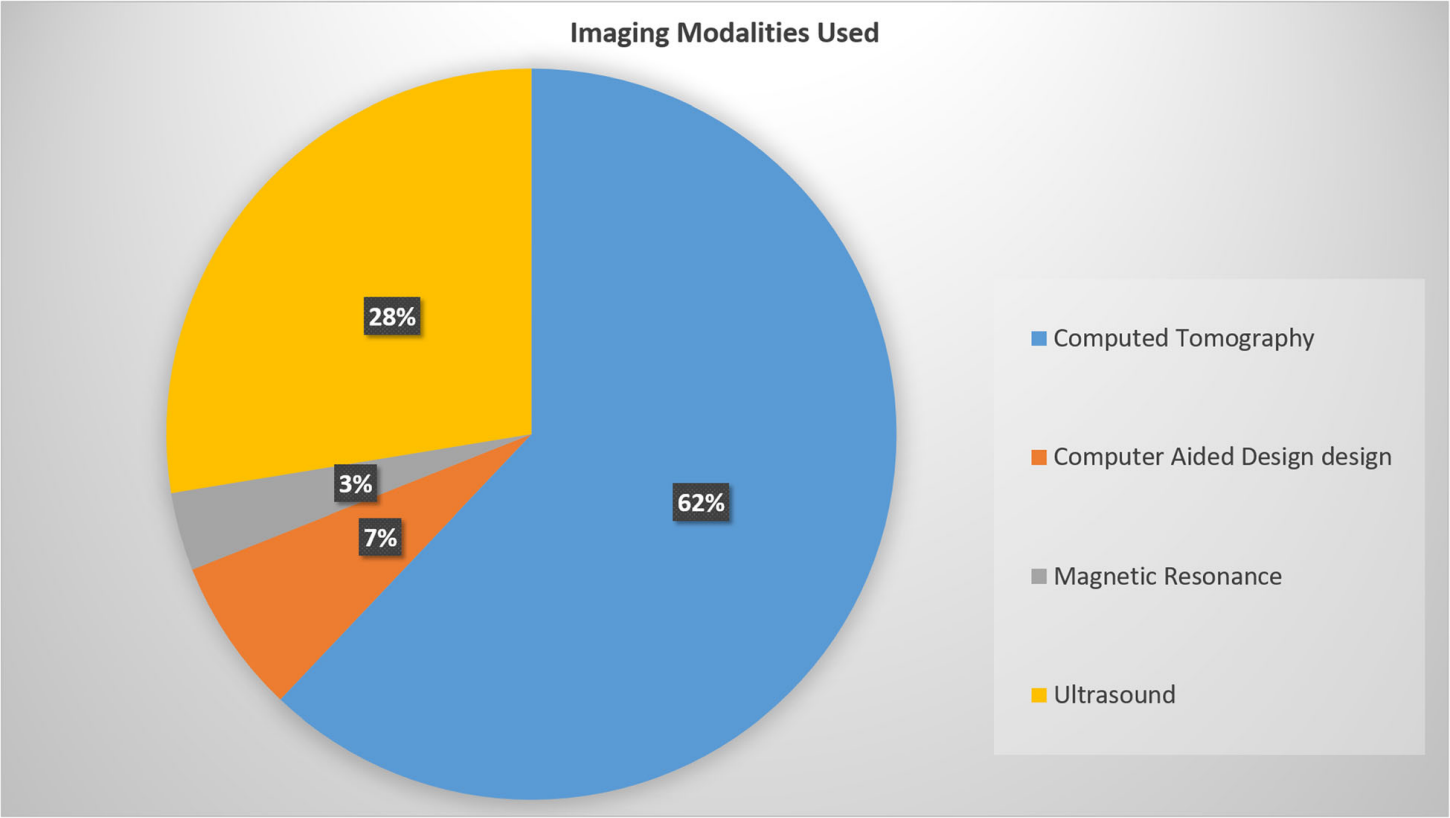
Frequency of use of different imaging modality providing the source data

A high-quality volume dataset with high resolution and no artefacts is required to allow for 3D printing. This can be acquired by common modern radiological imaging techniques provided that the proper reconstructions and protocols are applied. Computed tomography (CT) is the most common imaging modality providing image data for 3D printing in cardiac valve diseases (18 of 29 papers, 62%) [6–23], followed by US (8 papers, 28%) [9, 24–30], computer-generated models (computer-aided design) (2 papers, 7%) [31, 32], and magnetic resonance imaging (MRI) (1 paper, 3%) [34] (Fig. 2).

The quality of printed models is highly depending on the quality of the imaging dataset used. Cardiac motion and breathing artefacts have a negative impact on the segmentation and thus the printed volume. Typically, high-resolution scans are used in combination with electrocardiography gating, breath-hold, and/or respiratory gating [35]. In order to allow 3D printing of structures, they must have distinct tissue contrast in the imaging data [4].

In CT, commonly 0.75- to 1-mm slice thickness with a smoother kernel is used [4, 6, 35]. Scans with a higher resolution are less favourable since they introduce higher noise levels and require a more cumbersome segmentation process [35]. Some studies reported the use of multiphase acquisition during the cardiac cycle to ensure that the right phase can be reconstructed [20]. In MRI, standard cardiac imaging sequences can be used. However, the lower resolution of MRI in comparison with CT can hamper the production of good-quality 3D prints [35]. In US, the use of 3D scanning is required to obtain a proper 3D volume for segmenting the anatomical structures [24].

Regardless of the modality used to acquire the 3D datasets, structures of interest have to be segmented and translated into a surface model to enable 3D printing. Segmentation is the key process herein [33]. In some cases, the vessel wall is too thin to segment; extra thickness then should be added to the model since 3D printers have minimum thickness requirements [20].

The most commonly described tool for segmentation and creation of the STL file required for 3D printing is the Mimics/3-Matic software combination (Materialise, Leuven, Belgium). Secondly, SolidWorks (Dassault Systèmes SolidWorks Corporation, Vélizy-Villacoublay Cedex, France) is also used frequently. Less common are the 3D Slicer (Open source software package), AutoDesk Meshmixer (Autodesk, San Rafael, CA), and Vascular Modeling Toolkit (VMTK, Orobix, Bergamo, Italy).

All used packages have in common is that they allow to import the imaging data (according to the Digital Imaging and COmmunications in Medicine (DICOM) standard) from modalities such as CT, US, and MRI and transfer them to a 3D model. This model is realised by the segmentation of the structures of interest after which a surface representation is constructed. This surface reconstruction is commonly exported in STL format from the modeling software and loaded into the software of the 3D printer. This software allows to create and correct the model in order to ensure that it is printable and enables inclusion of required structures such as additional support material. After completion of the model, the data are resliced into print levels after which they can be sent to the printer to be manufactured.

## Printing materials

**Fig. 3:**
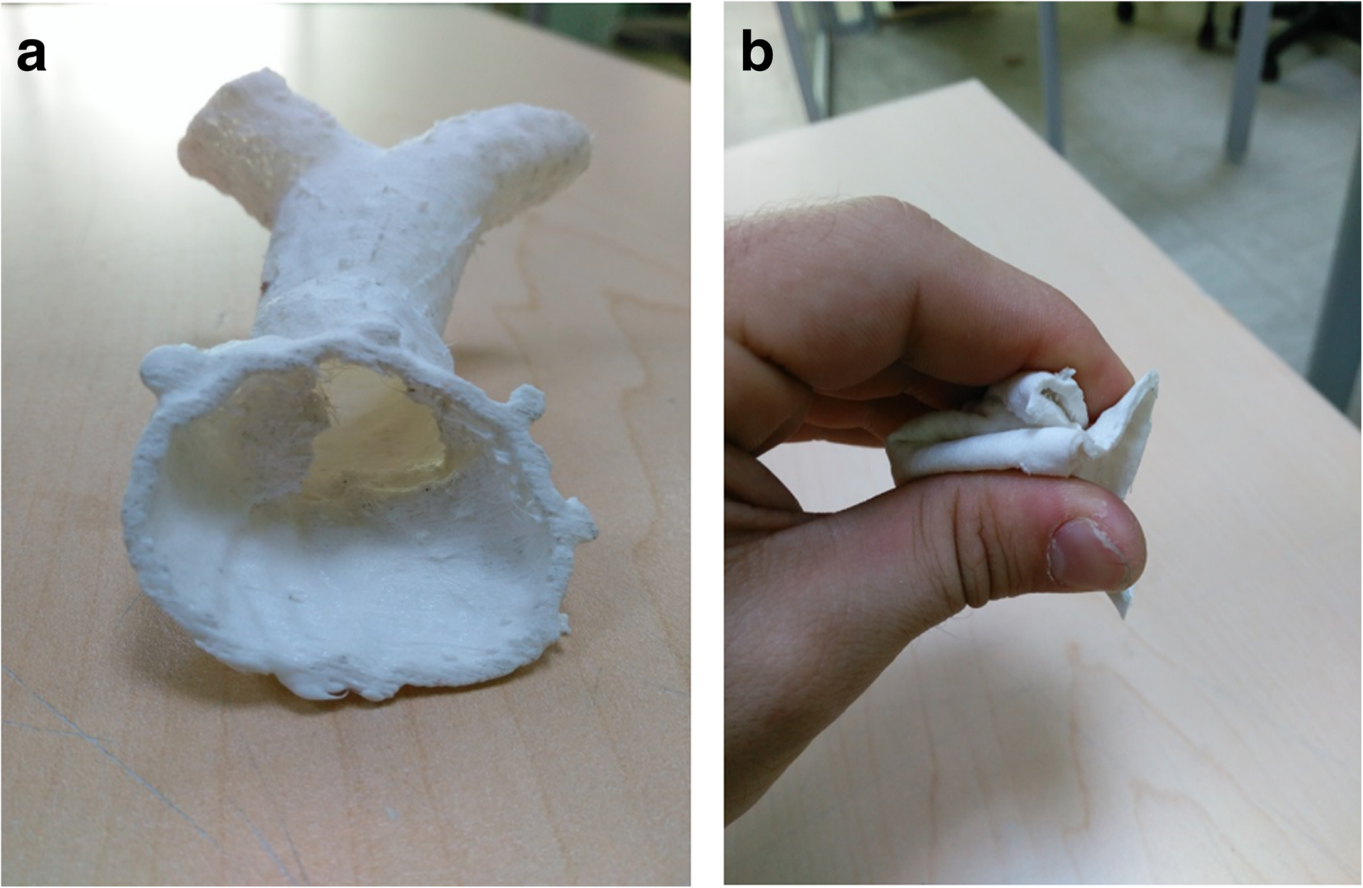
Example of the right ventricle outflow tract and main pulmonary artery print in flexible, non-transparent material in its normal (**a**) and squeezed (**b**) form

Although 3D printing is already known and used, one of the major areas of concern when printing cardiovascular structures such as the aorta, heart, and valves is the limited availability of usable printing materials to obtain objects with vessel-like properties. Traditional phantoms would be constructed with rigid models of resins or glass, but these are not useful when a more lifelike representation of the vessel wall is required. Therefore, the property requirements of printing materials for 3D printing of cardiovascular structures should take into consideration the flexibility of the material to mimic the vessel wall [11, 34] (Fig. 3) and the transparent nature of the materials [11] to allow for observation of instruments when inserted and visual inspection of internal structures.

The literature shows that acrylonitrile butadiene styrene or ABS (4/14, 29%) and TangoPlus FullCure 930 (5/14, 36%) (Stratasys Ltd., Eden Prairie, MN, USA) are the most commonly used materials (Table 1). TangoPlus FullCure 930 is a commercially available translucent rubber-like PolyJet photopolymer material. It can simulate different levels of hardness, elongation, and tear resistance. Because of the difficulty of direct printing in flexible materials, many papers describe a process in which print casts and molds are printed in other materials that are then dipped in, or coated with, silicone to obtain flexible vessels and valves with more accurate tissue properties [12, 27]. A challenge with this method is that it must either be possible to remove the silicone from the cast or mold after hardening or the cast or mold should be printed in a dissolvable material. Few examples of customised printers also exist that directly print with (sanitary) silicone [11, 31].

**Table 1:** 3D printing materials for cardiac valve replacement: intended uses and application areas

First author [reference number]	Intended use	Application area	Printing material	Post-treatment material
Abdel-Sayed [11]	Training	Trans-apical aortic valve replacement	Silicone	Silicone coating
Biglino [34]	Device testing	Material testing for cardiovascular application	TangoPlus FullCure 930	None
Fujita [12]	Preoperative planning	Transcatheter aortic valve implantation	Acrylonitrile butadiene styrene	Silicone coating
Fujita [13]	Retrospective procedure evaluation	Transcatheter aortic valve implantation	TangoPlus FullCure 930	Silicone coating
Fujita [14]	Preoperative planning	Transcatheter aortic valve implantation	Photopolymer resin	None
Izzo [7]	Preoperative planning	Transcatheter native mitral valve replacement	TangoPlus FullCure 930	None
Kalejs [31]	Device testing	Aortic valve replacement	Silicone	Silicone coating
Maragiannis [17]	Training	Aortic valve stenosis	TangoPlus FullCure 930	Silicone coating
Mashari [26]	Device testing	Mitral valve models	Moldstar 15 + Ecoflex 0030	Silicone coating
Ripley [20]	Preoperative planning	Transcatheter aortic valve implantation	Clear flexible resin	None
Sardari Nia [27]	Preoperative planning	Mitral valve intervention	Acrylonitrile butadiene styrene	Silicone coating
Vukicevic [9]	Training	Mitral valve intervention	TangoPlus FullCure 930	None
Witschey [28]	Preoperative planning	Mitral valve intervention	Acrylonitrile butadiene styrene	None
Owais [29]	Preoperative planning	Mitral annuli	Acrylonitrile butadiene styrene	None

**Fig. 4:**
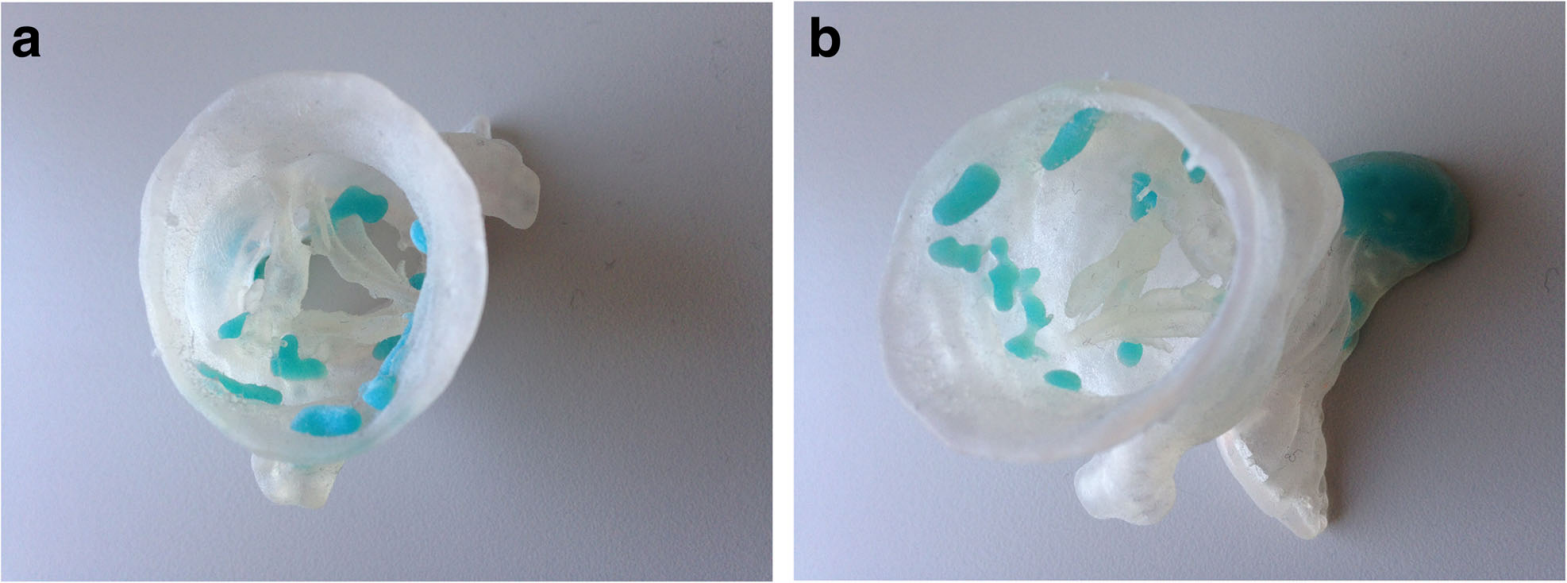
**a**, **b** Examples of prints of the aortic valve in a flexible transparent material with calcifications in blue non-flexible material

An additional requirement of specific interest in the case of the cardiac valves is the ability to print with multiple types of material to obtain flexible vessel walls and valves in combination with rigid calcified plaque deposits. Several reports with successful outcomes have been published using a transparent and flexible material for the vessel wall and valves combined with an opaque, rigid material for the calcified plaques [4, 7, 9, 17, 19, 20] (Fig. 4).

For example, Vukicevic et al. [9] 3D printed patient-specific mitral valves of three patients with multiple materials to evaluate trans-catheter mitral valve repair procedures. They performed biomechanical tests on different TangoPlus materials which were compared with the mechanical properties of the porcine mitral valve tissue to select the most appropriate TangoPlus material for a specific region of the 3D model. Different TangoPlus materials were used on different parts of the model in order to have the most realistic mechanical properties.

## Printing techniques

Several printing techniques exist. Among them, most frequently used and well known are *fused deposition modeling* (FDM), *STL*, *PolyJet*, and *laser sintering*. Details on these techniques have been described extensively in the literature [4, 5, 35, 36]. Based on the literature review performed, it is clear that STL is the preferred method for cardiac valve printing (40%), followed by FDM (30%). The preference for STL can be mainly explained by its ability to print more easily with flexible and transparent materials than other techniques.

In some cases, a dedicated setup was built to allow less conventional printing materials or printing hardware. One example setup in the literature was built with a syringe filled with (sanitary) silicone that was used to print a semi-transparent, flexible aortic root [31]. A high accuracy could be achieved (3.0% error along the *x*- and *y*-axes; 4.1% error along the *z*-axis). Although this was a very cheap solution, printing and post-processing time of the print were quite long (from 3 h and 20 min to 3 days).

## Time constraints

The current printing process involves the following steps:

1. imaging data acquisition;
2. segmentation of the anatomical structures;
3. export of the segmented structures to STL;
4. repair and improvement of the STL file;
5. re-slicing and preparing for printing (*e*.*g*., definition of support materials);
6. printing process;
7. post-printing (*e*.*g*., removal of support materials, silicone dipping).

The time required for each of these steps is not mentioned in all papers, and those that do mostly provide only a total processing time for steps 2–7 [11, 20, 24–26, 30, 34] (Table 2). The reported time ranges from 30 min for an FDM print of the mitral annulus [24] to 3 days for a dedicated FDM printer with a syringe filled with silicone followed by silicone dip coating for a simplified heart model [11]. Although the printing time is heavily depending on the size and complexity of the printed structure, experience shows that steps 6 and 7 are the most time-consuming. This especially holds in the case of molds and casts where extensive post-printing treatment is required, such as the application of the silicone (often with multiple coat dipping), and hardening of the material. Steps 2–5 are increasingly supported by dedicated software tools allowing more automation in the process and guided workflows to ensure a proper printing model.

**Table 2:** 3D printing for cardiac valve replacement: required time for different printed objects with various printing techniques

First author [reference number]	Printer	Print method	Post-treatment	Printed object	Time
Abdel-Sayed [11]	FDM syringe with silicone	FDM	Dip-coating with silicone	Simplified heart model	3 days
Biglino [34]	PolyJet	PolyJet	None	Descending aorta	12 h
Kalejs [31]	Fab@Home	FDM	Dip-coating with silicone	Aortic root model	200 min
Mahmood [25]	Objet260 Connex	PolyJet	NA	Mitral valve	90 min
Mahmood [24]	Makerbot Replicator 2X	FDM	None	Mitral annulus	30 min
Mashari [26]	Makerbot Replicator 2X	FDM	Silicone casting	Mitral valve	2–5 h
Muraru [30]	Formiga P110	Laser sintering	NA	Tricuspid valve	90–120 min
Ripley [20]	Form 1 Plus	SLT	NA	Aortic root	5 h
Owais [29]	Makerbot Replicator 2X	FDM	None	Aortic annulus	15 min

*FDM* fused deposition modeling, *SLT* stereolithography, *NA* not applicable

In general, the required time for the whole process of segmentation, data cleaning and preparation, and the printing itself greatly varies depending on the size of the printed object, the printing technique used, and the requirement for post-printing treatment.

## Possible printing issues

One of the issues with 3D printing is the accuracy of the 3D printed object in size and shape. The difference between the 3D printed object and imaging modality measurements should be minimal. The print accuracy is high using current printers and software with reported accuracies of a mean difference between the measurement in CT and of the print of − 0.34 mm ± 1.3 mm [20] and 0.7 mm ± 0.3 mm, respectively, without significant differences between the CT measurement and the actual print measurement [30]. Therefore, with careful design of the printing process, it is possible to print models that resemble the real anatomy and can be used for preoperative planning.

**Fig. 5:**
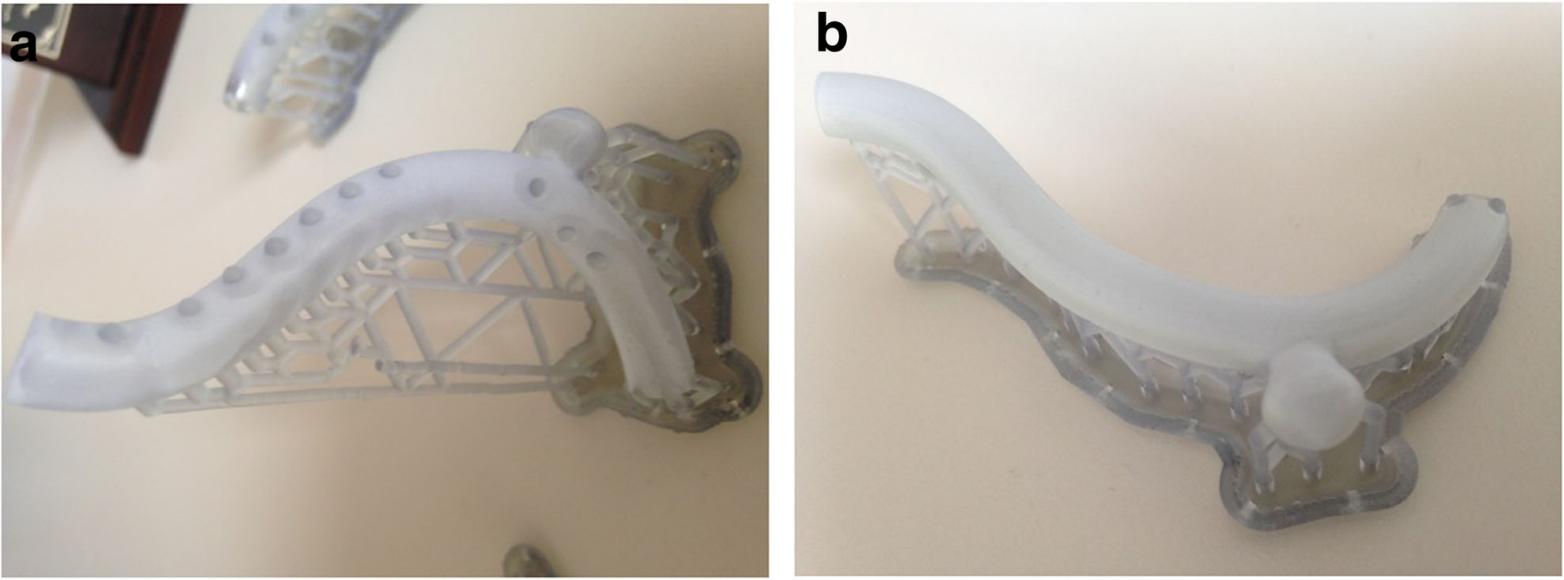
Example of a vessel print with support structures. When the vessel is printed in the anatomically correct orientation (**a**), it is running perpendicular to the printing surface and thus a lot of support material is required. When re-oriented and printed parallel to the printing surface (**b**), less support is required

Another issue is the removal of the support materials. Different from most other 3D printed models, the vascular models are complex in structure and must be hollow in order to gain access to the lumen with wires and devices. This can be achieved by either choosing a printing strategy not requiring support material (such as binder jetting) or by removing the support material after printing. A soluble support material can be used in a multiple material printer. In that case, the object usually has to be submerged into, for example, water to dissolve the support material [7]. The challenge is to fully remove all of the support material from the printed object manually during printing when the printing material for both object and support is similar. In vascular models, this can be quite challenging and complete removal of internal supports inside the artery structure can be difficult to obtain. Printing with supports also requires careful placement of the structure on the printing bed to minimise the negative effect of the support structures since the support required will be different with the orientation of the object (Fig. 5).

## Clinical applications

In the papers assessed, the main application areas found are preoperative planning (63%), training models (19%), device testing (11%), and retrospective procedure evaluation (7%).

### Preoperative planning

As stated before, recent years have shown an increase in minimally invasive cardiac surgery with the advent of procedures such as TAVR. One clear advantage of 3D printing is its utilisation in preoperative planning of such complex, minimally invasive cardiac surgery. Subsequently, the largest subgroup of the non-review papers (63%) concerns the use of 3D printing for preoperative planning. Of these, the subdivision between mitral and aortic valve replacement is approximately half and half.

When performing preoperative planning of TAVR, the printed anatomy ranges from only the aortic annulus [19, 20] or aortic arch/aorta [15, 21, 22] to more complex anatomical configurations with different anatomical structures in one print, including outflow tracts and heart chambers [12–14]. One study [16] not even only used 3D printed anatomy of the aorta but also 3D printed stent models.

The most crucial information for TAVR planning is the prediction of paravalvular aortic regurgitation (PAR). In one of the reported studies [20], the authors demonstrate the use of elastic 3D printed model for the prediction of PAR. It was done by using a light transmission test. The prosthesis was inserted into the 3D printed model, and the PAR was predicted based on a projection of light through the left ventricular outflow tract onto a thin film and captured with a digital camera. This correctly predicted PAR in six out of nine patients and absence of PAR in five out of seven patients.

### Training models

Transcatheter aortic valve replacement is a relatively new and fast growing therapeutic approach. Skills required to perform this kind of procedures are difficult to obtain, and a steep learning curve is perceived. Traditional training methods would require using animal models. This is costly and is gaining resistance by animal well-being organisations because of ethical considerations. Moreover, the logistics surrounding the use of animals or animal materials is rather cumbersome and complicated. A viable alternative can be found in an artificial heart model. However, this has a downside in that the variation in anatomy is limited and it often involves high manufacturing costs. 3D printing could solve this by providing an easy and relatively inexpensive method to obtain a wide variation of training samples that can be easily produced and replaced.

**Fig. 6:**
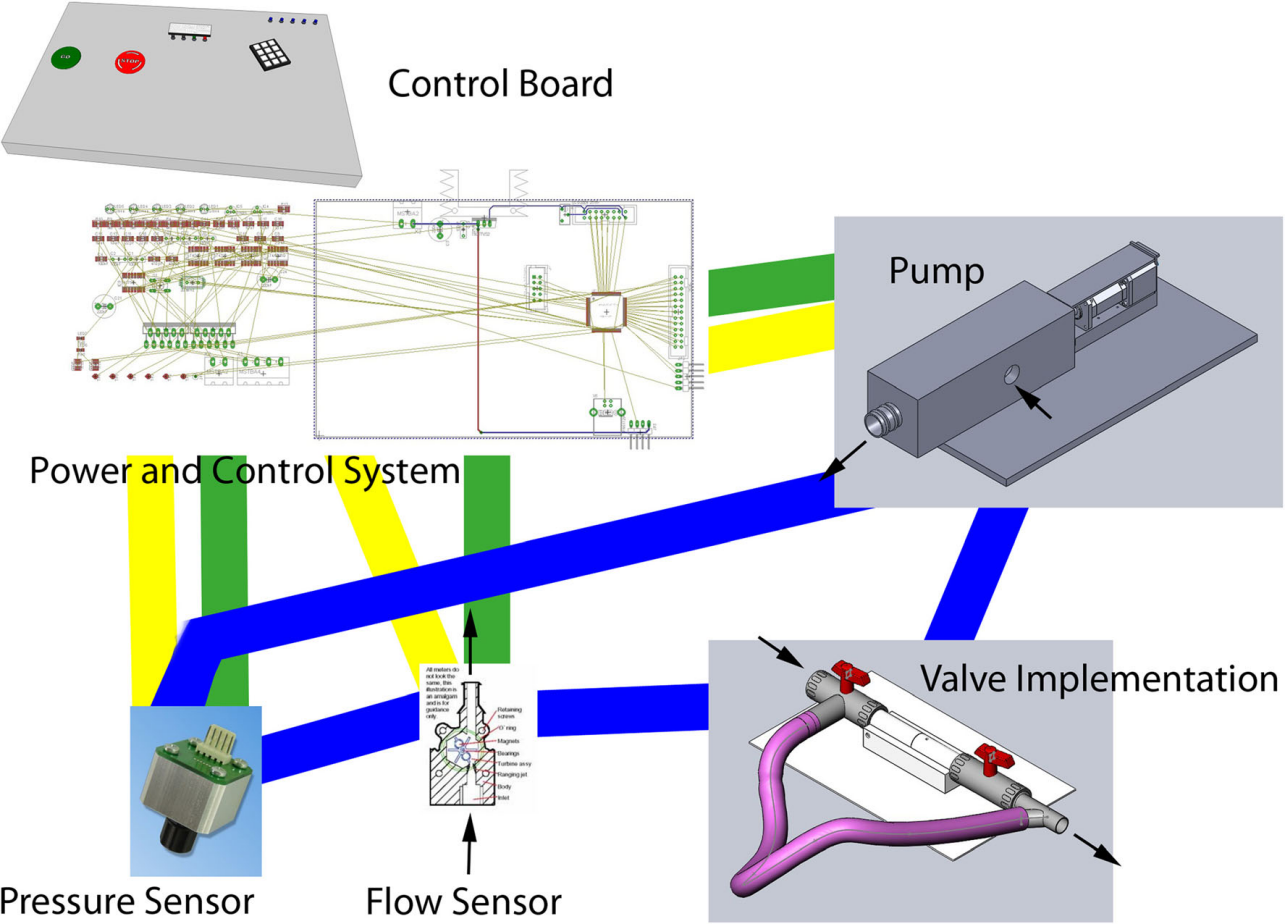
Sample schematic setup of an experimental environment to test valves. The 3D printed valve would be included in the valve implementation part inside the flow loop (blue lines)

The training models vary from simplified heart models [11] or geometrically designed aortic roots [31] to advanced flow models using pulsatile pumps allowing a more real-life simulation [5, 17] where devices can be introduced and deployed under lifelike conditions (Fig. 6). One study [32] described the printing of an MRI-compatible setup to allow scanning of mitral valves of pigs in the natural state. They achieved this by designing and 3D printing valve-specific mounting materials based on premortem US intra-valve measurements.

### Device testing

Device development and testing is also an important application of 3D printing. Biglino et al. [34] 3D printed models of the descending aorta with the same lumen dimension but with different wall thicknesses and did compliance tests. They used the distensibility knowledge to build a right ventricular outflow tract model, which was used to simulate the pulmonary valve replacement procedure for device testing. Kalejs and von Segesser [31] manufactured a real-life-size artic root model for testing valved stents. Mashari et al. [26] created a 3D model of the mitral valve from 3D US images of a patient who underwent a percutaneous MitraClip operation, a minimally invasive procedure to reduce the mitral regurgitation. The model was then deployed in the pulse-duplicator chamber filled with a blood-mimicking fluid for hemodynamic testing.

## Discussion

Novel techniques such as 3D printing are investigated by different research groups for specific clinical questions, and they all explore the technical requirements and shortcomings of the technique. While the topic is current, *the actual clinical benefit of 3D printing yet remains to be proven*. However, technical developments are ongoing and the implementation of 3D printing for cardiac valve treatment is one of the more promising clinical application areas. However, the application of 3D printing in cardiac valve replacement introduces additional requirements on the used printing material and the nature of the printed structure. This review shows that, with the advent of new flexible and transparent materials and higher accuracy of 3D printers, an accurate representation of the cardiac anatomy can be obtained. This 3D printed representation of the anatomy can already be used in a variety of applications, especially for training purposes.

It has been shown that for accurate 3D printing of anatomy or pathology, correct segmentation is of vital importance. Although some of the segmentation work is currently automated, the input of experts is still needed for validation and correction. Any small mistake in the segmentation process could lead to erroneous prints. This could lead to the failure of the printing process itself, but also to a 3D printed structure that is not accurate that, in turn, could be harmful to the patient when used for preoperative planning. Thus, the segmentation step should be conducted and/or supervised by an expert in the anatomy being segmented, and quality control of the source data should be in place. Also, the segmentation tools should be validated and approved for clinical use. Although the majority of the reviewed papers show the use of a validated commercial software tool, researchers may still rely on freeware and open-source software which could hamper reliability of the segmentation, 3D model construction, and finally the printing result.

In this review, we aimed to explore the options, challenges, and possibilities of the 3D printing in the field of cardiac valve replacement in order to give an insight into the current state of the art and development in this specific area of 3D printing. The low number of papers found on this topic demonstrates its experimental nature. However, the published papers do show the progress made in the past years allowing for clinical application. This clinical application is currently mainly in training and education, but the literature is promising for actual patient-specific clinical applications.

Current technology allows for an accurate printing of cardiac anatomy in materials that resemble the properties of the actual heart and vessels. The application of 3D printing in valve replacement planning could therefore provide new insights into many different ways for the different stakeholders [33]. It can provide better insight into the anatomy and allow preoperative training for the treating physician [5]. For the patient, it can provide more insight into the disease and treatment options [4, 35]. For the manufacturer, it allows easier preclinical testing of new devices or instruments. And finally, for the educators, it can provide a wide variety of anatomical and pathological examples that would normally be unavailable.

## Abbreviations

3D: Three-dimensional
CT: Computed tomography
FDM: Fused deposition modeling
MRI: Magnetic resonance imaging
PAR: Paravalvular aortic regurgitation
STL: Stereolithography
TAVR: Transcatheter aortic valve replacement
TMVR: Transcatheter mitral valve replacement
US: Ultrasound

## References

[1] Sutherland J, Belec J, Sheikh A et al (2018) Applying modern virtual and augmented reality technologies to medical images and models. J Digit Imaging. 10.1007/s10278-018-0122-7 [Epub ahead of print] 10.1007/s10278-018-0122-7 PMC6382635 30215180

[2] Mitsouras D, Liacouras P, Imanzadeh A (2015). Medical 3D printing for the radiologist. Radiographics.

[3] Binder TM, Moertl D, Mundigler G (2000). Stereolithographic biomodeling to create tangible hard copies of cardiac structures from echocardiographic data. J Am Coll Cardiol.

[4] Giannopoulos AA, Steigner ML, George E (2016). Cardiothoracic applications of 3-dimensional printing. J Thorac Imaging.

[5] Vukicevic M, Mosadegh B, Min JK, Little SH (2017). Cardiac 3D printing and its future directions. JACC Cardiovasc Imaging.

[6] Dankowski R, Baszko A, Sutherland M (2014). 3D heart model printing for preparation of percutaneous structural interventions: description of the technology and case report. Kardiol Pol.

[7] Izzo RL, O’Hara RP, Iyer V et al (2016) 3D printed cardiac phantom for procedural planning of a transcatheter native mitral valve replacement. Proc SPIE Int Soc Opt Eng 9789 10.1117/12.2216952 PMC5467736 28615797

[8] Little SH, Vukicevic M, Avenatti E, Ramchadani M, Barker CM (2016). 3D printed modeling for patient-specific mitral valve intervention: repair with a clip and a plug. JACC Cardiovasc Interv.

[9] Vukicevic M, Puperi DS, Jane Grande-Allen K, Little SH (2017). 3D printed modeling of the mitral valve for catheter-based structural interventions. Ann Biomed Eng.

[10] Wang DD, Eng M, Greenbaum A (2016). Predicting LVOT obstruction after TMVR. JACC Cardiovasc Imaging.

[11] Abdel-Sayed P, Kalejs M, von Segesser LK (2009). A new training set-up for trans-apical aortic valve replacement. Interact Cardiovasc Thorac Surg.

[12] Fujita B, Kütting M, Scholtz S (2015). Development of an algorithm to plan and simulate a new interventional procedure. Interact Cardiovasc Thorac Surg.

[13] Fujita B, Kütting M, Seiffert M (2016). Calcium distribution patterns of the aortic valve as a risk factor for the need of permanent pacemaker implantation after transcatheter aortic valve implantation. Eur Heart J Cardiovasc Imaging.

[14] Fujita T, Saito N, Minakata K, Imai M, Yamazaki K, Kimura T (2017). Transfemoral transcatheter aortic valve implantation in the presence of a mechanical mitral valve prosthesis using a dedicated TAVI guidewire: utility of a patient-specific three-dimensional heart model. Cardiovasc Interv Ther.

[15] Gallo M, D’Onofrio A, Tarantini G, Nocerino E, Remondino F, Gerosa G (2016). 3D-printing model for complex aortic transcatheter valve treatment. Int J Cardiol.

[16] Hernández-Enríquez M, Brugaletta S, Andreu D (2017). Three-dimensional printing of an aortic model for transcatheter aortic valve implantation: possible clinical applications. Int J Cardiovasc Imaging.

[17] Maragiannis D, Jackson MS, Igo SR (2015). Replicating patient-specific severe aortic valve stenosis with functional 3D modeling. Circ Cardiovasc Imaging.

[18] O’Neill B, Wang DD, Pantelic M (2015). Transcatheter caval valve implantation using multimodality imaging: roles of TEE, CT, and 3D printing. JACC Cardiovasc Imaging.

[19] Qian Z, Wang K, Liu S (2017). Quantitative prediction of paravalvular leak in transcatheter aortic valve replacement based on tissue-mimicking 3D printing. JACC Cardiovasc Imaging.

[20] Ripley B, Kelil T, Cheezum MK (2016). 3D printing based on cardiac CT assists anatomic visualization prior to transcatheter aortic valve replacement. J Cardiovasc Comput Tomogr.

[21] Schmauss D, Schmitz C, Bigdeli AK (2012). Three-dimensional printing of models for preoperative planning and simulation of transcatheter valve replacement. Ann Thorac Surg.

[22] Schmauss D, Haeberle S, Hagl C, Sodian R (2015). Three-dimensional printing in cardiac surgery and interventional cardiology: a single-centre experience. Eur J Cardiothorac Surg.

[23] Sodian R, Schmauss D, Markert M (2008). Three-dimensional printing creates models for surgical planning of aortic valve replacement after previous coronary bypass grafting. Ann Thorac Surg.

[24] Mahmood F, Owais K, Montealegre-Gallegos M (2014). Echocardiography derived three-dimensional printing of normal and abnormal mitral annuli. Ann Card Anaesth.

[25] Mahmood F, Owais K, Taylor C (2015). Three-dimensional printing of mitral valve using echocardiographic data. JACC Cardiovasc Imaging.

[26] Mashari A, Knio Z, Jeganathan J (2016). Hemodynamic testing of patient-specific mitral valves using a pulse duplicator: a clinical application of three-dimensional printing. J Cardiothorac Vasc Anesth.

[27] Sardari Nia P, Heuts S, Daemen J (2017). Preoperative planning with three-dimensional reconstruction of patient’s anatomy, rapid prototyping and simulation for endoscopic mitral valve repair. Interact Cardiovasc Thorac Surg.

[28] Witschey WR, Pouch AM, McGarvey JR (2014). Three-dimensional ultrasound-derived physical mitral valve modeling. Ann Thorac Surg.

[29] Owais K, Pal A, Matyal R (2014). Three-dimensional printing of the mitral annulus using echocardiographic data: science fiction or in the operating room next door?. J Cardiothorac Vasc Anesth.

[30] Muraru D, Veronesi F, Maddalozzo A (2017). 3D printing of normal and pathologic tricuspid valves from transthoracic 3D echocardiography data sets. Eur Heart J Cardiovasc Imaging.

[31] Kalejs M, von Segesser LK (2009). Rapid prototyping of compliant human aortic roots for assessment of valved stents. Interact Cardiovasc Thorac Surg.

[32] Stephens SE, Liachenko S, Ingels NB, Wenk JF, Jensen MO (2017). High resolution imaging of the mitral valve in the natural state with 7 tesla MRI. PLoS One.

[33] Vaquerizo B, Theriault-Lauzier P, Piazza N (2015). Percutaneous transcatheter mitral valve replacement: patient-specific three-dimensional computer-based heart model and prototyping. Rev Esp Cardiol (Engl Ed).

[34] Biglino G, Verschueren P, Zegels R, Taylor AM, Schievano S (2013). Rapid prototyping compliant arterial phantoms for in-vitro studies and device testing. J Cardiovasc Magn Reson.

[35] Giannopoulos AA, Mitsouras D, Yoo SJ, Liu PP, Chatzizisis YS, Rybicki FJ (2016). Applications of 3D printing in cardiovascular diseases. Nat Rev Cardiol.

[36] Meier LM, Meineri M, Qua Hiansen J, Horlick EM (2017). Structural and congenital heart disease interventions: the role of three-dimensional printing. Neth Heart J.

